# #WeAreNotWaiting DIY artificial pancreas systems and challenges for the law

**DOI:** 10.1111/dme.14715

**Published:** 2021-10-21

**Authors:** Rachael Dickson, Jessica Bell, Amber Dar, Laura Downey, Victoria Moore, Muireann Quigley

**Affiliations:** 1 University of Birmingham Law School, Birmingham, UK; 2 University of Warwick Law School, Coventry, UK; 3 The University of Manchester School of Social Sciences, Manchester, UK

**Keywords:** clinical diabetes, devices, medical law, patients

## Abstract

Commercial hybrid closed-loop systems are becoming more readily available, yet the number of DIY artificial pancreas system (DIY APS) users continues to rise. These DIY systems have not gone through the usual regulatory approvals processes, and, thus, present a number of legal difficulties for a number of actors, including clinicians, parents who build DIY APS for their children, and users themselves. These issues have so far received insufficient attention. Due to the complex constellation of actors involved in both development of DIY APSs and in its deployment, it is not currently clear who, and to what extent, different parties might (successfully) be held liable if something goes wrong. Despite this uncertainty, unless and until clearer guidance is issued by relevant bodies, or a case appears before the courts which clarifies the situation, existing legal principles apply. In this article, we examine some of these to shed light on how the law would likely be applied if harm were to result from the use of a DIY APS.

## 1 Introduction

Despite the recent increase in commercial hybrid closed-loop systems developed, the number of DIY artificial pancreas system (DIY APS) users continues to rise.^1^ There are likely a number of reasons for this, but one reason is that system development does not necessarily translate into user availability. For instance, in the United Kingdom, only four commercial systems have gained the requisite regulatory approvals^2^ and not all of these are available on the NHS or available to all persons with diabetes (PWDs) depending on their geographical location.^3^ Another reason is the fact that at least some DIY users prefer the functionality and customisability offered by the DIY systems, meaning they won’t be switching to the new commercial systems.^4^ As such, not only do DIY APSs seem here to stay for the foreseeable future, but new DIY systems (e.g. FreeAPSX), building on the success of those already available (OpenAPS, AndroidAPS, and Loop), are entering the fray.

These DIY systems consist of three components: a smartphone/small computer which runs an algorithm and collects data, a continuous glucose monitor (CGM) to provide glucose readings and an insulin pump to deliver insulin. Once connected, the resulting system automatically calculates and delivers insulin doses.^5^ Users self-reported benefits include improved blood-glucose management and decreased anxiety around hypoglycaemia.^6^

The continued growth in the use of DIY systems notwithstanding the fact remains that they have not been through the usual regulatory approvals processes. DIY APSs, thus, present a number of legal difficulties—for developers, healthcare professionals, and users—which so far have received insufficient attention. The servers which host the code and instructions for building the different DIY APSs are located outside both the UK and the EU. As two of us have noted elsewhere, the result is that “both legally and in practice, DIY APSs fall through a regulatory gap.”^5^ This means that they are not captured by usual approvals and manufacturer registration processes.^1^ Yet, whilst there is broader regulatory uncertainty about the status of DIY APSs which needs to be addressed, in current practice, the legal obligations and responsibilities of clinicians and other actors vis-à-vis DIY APS largely fall under the law of negligence.

The law of negligence aims to provide remedies for persons who have suffered harm as a result of someone else’s failure to take proper care in dispensing their duties. However, there are a number of reasons why it may be unclear whose fault or failure (if anyone’s) is responsible for harm suffered in the DIY APS context. The initial and continuing development of DIY APSs is a collaborative community effort, driven and supported by highly motivated, expert, and technologically skilled people. In many situations, determining who has contributed to particular parts of technological projects may not be much of a problem. However, DIY APS development involves numerous actors at different stages. Some of these stages include the development of algorithms and code, testing the system, making the tools available to others to build the system and the implementation and running of the final built system. Once built, users are likely to require some degree of involvement from their healthcare professionals, even if that involvement is simply the continued prescribing of relevant components.

**Figure 1:**
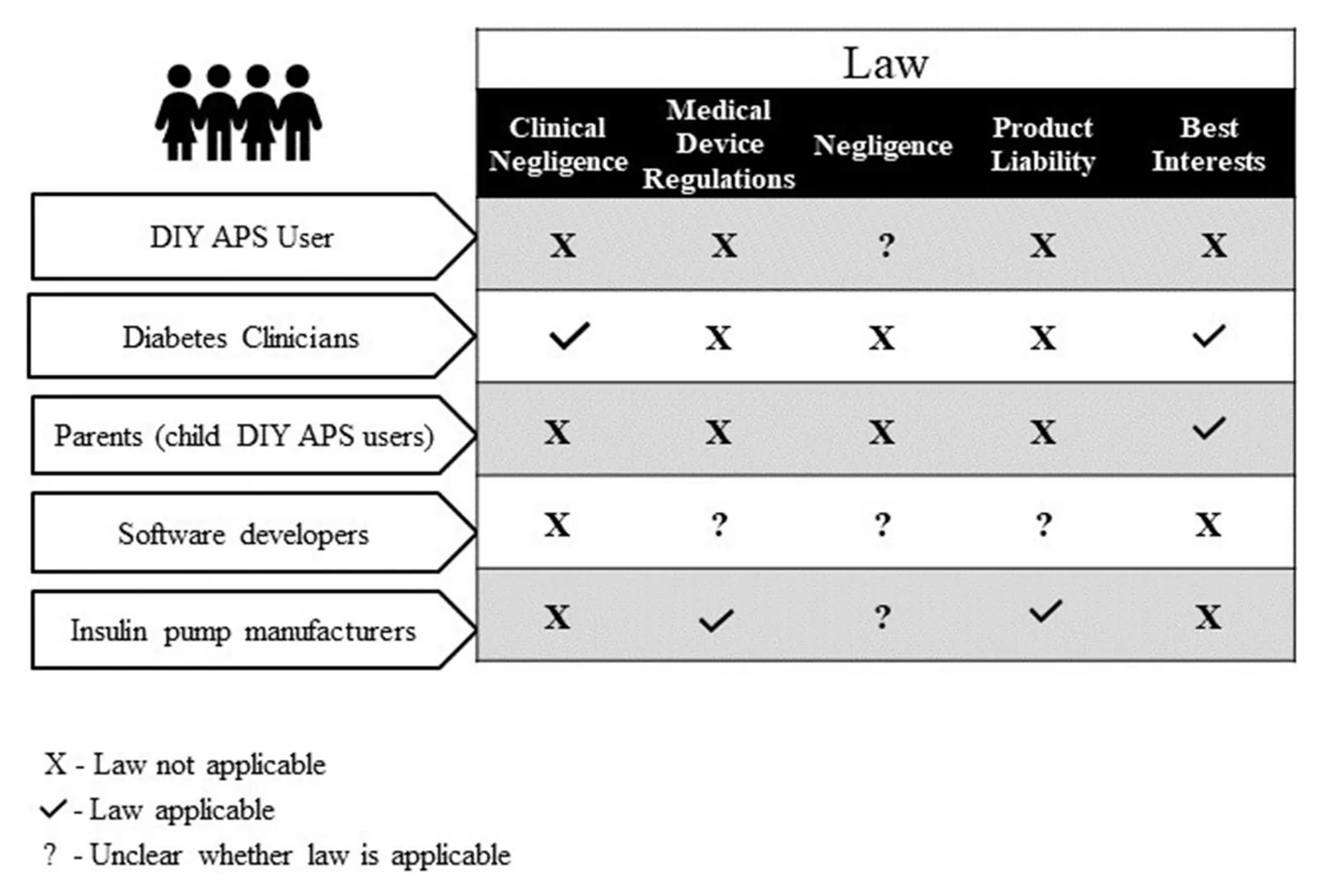
Summary of different actors and relevant law [Correction added on 22 December 2021 after first online publication: Data in Software developers row have been updated in this version.]

Due to the complex constellation of actors involved in both the development of a DIY APS and in its deployment, it is not currently clear under UK law^2^ who may be held liable if something goes wrong. In this article, we look at some of the key actors involved in the DIY APS ecosystem and broadly outline issues regarding potential liability. Focusing on clinicians, parents of child loopers, insulin pump manufacturers and DIY APS software developers, we ask who, if anyone, might be liable for the harms arising. The law is, of course, a lot more complex than we can convey in this short article, but we hope that what we have written will prove helpful for those interested in the law and DIY APSs. For a summary of the different actors and relevant areas of law see Figure 1 above.

## 2 Clinician Liability and Adult Loopers

We recognise that clinicians may be concerned that by being involved in a PWD’s use of a DIY APS, they may be held liable for any subsequent harm that the person experiences.^7,8^ When considering whether or not they could be liable, it is important to recognise that, even though DIY APS is an emerging technology, the actions of the clinician are subject to the usual law of negligence.^9^ In many respects, this is no different to all other aspects of a clinician’s practice. This means that it must be proven that the clinician breached their duty of care, which resulted in harm to the patient. In considering the possible liability of clinicians, the first question we address here is, could a clinician be liable for *failing* to tell the PWD about DIY APS as a treatment option? We then turn our attention to whether a clinician might be liable for *discussing* DIY APS with their patients. And finally, we ask, what about if clinicians were to *prescribe* the relevant technology/system components?

In answer to our first question, doctors must inform patients about any ‘reasonable alternative treatment’ when discussing their treatment options.^9^ This has been defined by the courts as being sensitive to the circumstances of any given case (including consideration of the patient, their condition, and their prognosis at the time) and needing to be within the knowledge of a reasonably competent clinician.^10^ It is, therefore, unlikely *at the present time* that not telling a PWD about DIY APS would be seen as negligent since there is still a dearth of evidence in medical journals about this as a treatment option. However, it might be that a competent diabetes specialist would be expected to have heard of, and have knowledge about, DIY APSs, given their increasing popularity amongst PWDs.

With regard to our second question about discussing DIY APS with a patient, we first need to consider what that discussion might look like. This is because in some cases, clinicians might simply be providing information about DIY APS, but without recommending it or advising on its use. In other circumstances, a discussion might incorporate advising or recommending, which could count as prescribing.^5^ Providing information is unlikely to lead to liability issues. Indeed, the most recent iteration of the GMC’s ‘consent guidance’ says that the exchange of information between doctor and patient is central to good decision-making.^11^

Nevertheless, if a clinician were to tell a PWD about a DIY APS, perhaps with a view to the person considering this as a treatment option, they would need to ensure patients are appropriately informed of the risks. As confirmed by the Supreme Court in the 2015 case of *Montgomery*, doctors have a duty to take reasonable care to ensure patients are aware of any material risks involved in their treatment.^9^ In this particular case, a woman with type one diabetes claimed she ought to have been informed of the risk of shoulder dystocia occurring during the birth of her child. The doctor had not informed her of this risk as she had deemed it to be a very small risk. The child was born with severe disabilities as a result of experiencing shoulder dystocia, and the woman argued she would have opted for a caesarean section had she known of the risk. Although the risk had been small, the Court determined that it was nevertheless a material risk which the patient should have been informed of.

A ‘material risk’ reflects a variety of factors—such as the nature of the risk, the effect its occurrence would have upon the patient’s life, the importance to the patient of the treatment’s benefits, and the availability and risks of any alternatives. This information must be provided to the patient in an understandable manner. Based on these requirements, if doctors provide information on DIY APS to a PWD in a manner that makes that person aware of any material risks, and ensures that they understand the information, it is unlikely they would be found to be negligent.

We now turn to consider a situation where a doctor advises a PWD on their use of a DIY system. If a doctor makes a negligent statement, which is then relied upon by the PWD, the doctor might be held liable for any resulting harm.^12^ This happened in one case where a patient alleged that a doctor had failed to take reasonable care to ensure that the information given regarding a vasectomy was correct. The procedure had been unsuccessful, but the doctor incorrectly advised the couple that the husband’s sperm count was negative and contraceptives were no longer necessary.^13^ The claimants, therefore, argued that a subsequent pregnancy and birth were the direct and foreseeable result of the doctor’s negligence. Applying this to the DIY APS context, it is possible that a doctor could be found negligent if they provide incorrect or misleading advice which their patient then relies upon and which results in harm. This may occur, for example, if a DIY APS user seeks technical input from their doctor, who gives advice (however, well-intentioned) based on an erroneous understanding of the technology. Given the emerging state of the technology and user base, it is not currently clear what doctors would be expected to know about DIY APS. What is clear, however, is that they should be scrupulously honest with patients about the *limits of their knowledge* and explain if they are unsure or cannot advise them appropriately.^12^

With regard to our third question about liability arising from prescribing the relevant components (e.g. insulin pumps or pump consumables), clinicians would only be liable if they breach their duty of care in a manner which results in reasonably foreseeable harm to the patient. Doctors are not negligent if they act in accordance with accepted practice by other medical practitioners skilled in that particular field, providing the practice can withstand logical analysis.^14^ This means that a clinician would likely only be found negligent if no other responsible clinician would have acted in a similar manner and that the decision to do so was illogical. However, the exact circumstances will be relevant, and there may be circumstances concerning a particular patient that would mean a clinician’s prescribing choices may be entirely illogical and inappropriate. Consider, for example, a PWD who is already using the relevant technology and says that they intend to close the loop. A clinician who then decides to withhold prescriptions from that patient so that they cannot build their DIY APS could be found to be negligent if this results in harm to the patient.

A different scenario might be where a PWD requests that specific components are prescribed so that they can create a DIY system. Doctors do not have to prescribe a treatment which they do not think is in the patient’s clinical interests.^15^ Whether a treatment is appropriate might be informed by NICE guidelines on patients who meet the criteria for CGMs and pumps. In such a scenario, we would not anticipate that a clinician’s actions would amount to negligence if they deliver the expected standards of care for a patient with type one diabetes.

If a DIY APS user does not inform their doctor that they are using a DIY APS, or is not truthful and says they are not using such a system, it is very unlikely that a doctor could be held liable for any harm. Even if a doctor is found to be liable to some extent, a finding of contributory negligence might work against the claimant if the claimant’s own negligence has contributed to their injury. Although it is not common in the UK, patients receiving medical treatment can be found legally responsible for any actions that may have contributed to their injury or made their existing medical condition worse.^16^ A recent road traffic accident case confirmed that omissions (by healthcare professionals) should be apportioned less liability for contributory negligence than positive actions by the harmed individual, which could influence potential DIY APS cases.^17^ Considering the proactive role that DIY APS users are taking in building, managing and maintaining their own DIY systems, it would not be unreasonable to suggest that patients may be partially responsible for any resulting harm.

## 3 What About The Children?

A further question regarding DIY APSs use relates to the legal implications for parents who build DIY APSs for their children and whether additional ethical and legal responsibilities arise for healthcare professionals treating children whose parents have built and use DIY APSs.

The difficulty in defining ‘innovative treatment’,^18^ and lack of legal regulation for such treatment, is of particular concern for cases that involve parents building a DIY APS for children with diabetes. It remains necessary to rely upon professional standards and guidelines (which are not legally binding),^19^ and to apply general principles of civil^3^ and criminal law to provide some form of a legal regulatory framework for innovative treatment.^15^ This can raise additional concerns and stress for children, their families and healthcare teams managing a child’s medical care and treatment.

When a young child is the patient, a parent or carer who has parental responsibility will make decisions on their behalf.^20^ Whether or not decisions regarding DIY APSs fall within acceptable bounds of parental discretion regarding their child’s medical treatment has not been tested in court. It has been suggested that if parents assume a risk using a DIY system, a doctor’s duty of care is discharged by discussing risks (namely the risks of using devices with modified functionality), including how these risks are likely to impact any care the doctor and healthcare team can now provide.^21^ However, it should be noted that GMC guidance requires that doctors always act in the best interests of children and young people. As such, the circumstances of the particular child and their family will determine whether and to what extent their duty of care is discharged through a discussion of risks.^21^

Situations may arise where healthcare professionals disagree with a parent’s choice to build and use a DIY APS for their child, or are concerned about how the parents intend to fund and safely use an unregulated device. Whilst there will be particular challenges and concerns with any new technologies in healthcare, case law provides some indication of the courts’ approach in circumstances where there is a disagreement between parents and healthcare professionals on medical grounds. The first thing to note in this respect is that a landmark case, *Glass v United Kingdom*, confirmed that the healthcare treatment decisions regarding dependent children are to be made by their parents, not healthcare professionals, and where there is a disagreement (which cannot be resolved between the parties) about viable treatment options, decisions are to be made by the court.^22^ In this particular case, it was claimed that the rights of both mother and child had been violated by putting a ‘do not resuscitate’ (DNR) order on the child’s hospital case notes without the consent of his mother.

Cases involving the medical treatment of children can end up in court where suffering or harm to the child has resulted (or might result) from (1) disagreements where parents have acted/are intending to act against medical advice or (2) failure of parents to seek medical advice when necessary and appropriate to do so. For instance, in a recent case that deals primarily with the issue of innovative treatment, the main concerns stemmed from the differences between the parents’ account and the hospital staff’s account of what happened at the hospital after the disagreement.^23^ In this case, the parents and clinicians did not agree on the type of radiotherapy to be administered to the child—the hospital advised conventional radiotherapy and the parents wanted a new type—known as proton therapy—which was available in Prague, but not the UK. The UK clinicians and Hospital Trust said they did not oppose the treatment the parents wanted, but needed to ensure the treatment could be arranged and funded and that the child could be safely transferred. In the DIY APS context, the exact approach of the courts would likely be determined by the nature of the disagreement.

Importantly, recent case law highlights that good communication between healthcare professionals and those with parental responsibility is crucial to avoid matters reaching the courts. For example, in a hypothetical scenario where a doctor is concerned about the risk of harm to a child because the parents may act against the advice of the doctor, the best course of action would be to maintain an open dialogue and good communication with the parents as much as possible. This would include non-judgmentally explaining the risks posed.^20^ In such circumstances, unless the risk of harm provides sufficient justification, an application to court is likely to have a negative impact on the child and their family, and on the relationship of trust between the healthcare team and the family.^20^

Turning now to those with parental responsibility, there are specific statutory provisions concerning this, as well as the welfare of the child and the application of a ‘best interests’ approach when the welfare of a child is in question. Relevant statutory frameworks broadly outline and define the concept of parental responsibility.^24^ In making any decisions about a child or a child’s upbringing, the welfare of the child is the court’s paramount consideration.^22^ Here, the case law provides guidance on how the best interests of patients have been assessed in circumstances where innovative or experimental treatment is available.^25^

Broadly, best interests must be assessed ‘in the widest possible way to include the medical and non-medical benefits and disadvantages, the broader welfare issues [of individual patients]…their abilities, their future with or without treatment, the views of the families and the impact of refusal of the applications’.^23^ All such matters have to be ‘weighed up and balanced in order for the court to come to a decision in the exercise of its discretion’.^23^ This suggests that the circumstances of parents would be closely scrutinised to determine whether or not there was sufficient justification for a parent’s decision to choose a DIY APS and if this was in the best interests of the child.^25^

Whilst parents may be able to justify the adoption of a DIY APS for their child, guidance is needed to provide clarification on whether, and in what circumstances, it can be in the best interests of a child to be given an unregulated, novel treatment that has been created by members of the public rather than medical specialists. The benefit of DIY APS over and above the approved alternative—in terms of it being expected to achieve equally good or better outcomes—would need to be justified and arguments/evidence in this respect deemed compelling. Whilst case law can provide some guidance on assessing best interests and decision-making about children, updated ethical guidance and regulatory reform could better inform clinicians and those with parental responsibility. This could in turn reduce the likelihood of disagreement and conflict between parents and healthcare professionals on medical grounds.

## 4 Insulin Pump Manufacturers and Software Developers

Beyond the PWD (adult or child) and the clinician, there are numerous other actors involved in DIY APSs processes. The two main groups which could potentially be held liable for any harm arising are: (1) Manufacturers of insulin pumps used by those who build a DIY APS and (2) software developers responsible for developing the requisite algorithms and code (often DIY APS users themselves). We ask two questions here: Can either manufacturers or developers be held responsible if a DIY APS malfunctions? And do either owe users a duty of care which could result in a claim in negligence?^4^

Let us deal with the pump manufacturers first. Generally speaking, manufacturers can be held strictly liable for any damage caused by a defective product under the auspices of product liability law.^26^ Although manufacturers of insulin pumps are straightforwardly the ‘producers’^26^ of insulin pump ‘products’,^26^ they would likely dispute any claim of liability for harm resulting from pumps used for a DIY APS. After all, in the main, these insulin pumps are used for purposes that are not originally intended by the manufacturer. One point that runs counter to this is the fact that certain manufacturers knowingly leave open their pumps’ communication protocols, thus enabling them to be used for looping. The result of this could be that these pumps would be viewed by the courts as being ‘defective’.^26^ However, since pump manufacturers commonly warn against the risks associated with off-label use, such warnings could be viewed as being sufficient to discharge their legal obligation in this respect.^5^

From a negligence standpoint, a strong argument can be made that the effort which DIY APS users expend in setting up their systems means that they have consented to, or voluntarily accepted, the potential risk of harm. Both consent or voluntary acceptance could be a valid defence to any claim of pump manufacturer negligence. Even though a door has been left open by certain pump manufacturers which enables ‘looping’, significant further steps are taken by the user to create their DIY APS.

Turning to software developers, difficulty arises here because software as a medical device (which is what a DIY APS is) is a poor fit for current law and regulation.^27^ This is because the current regulatory framework was designed with tangible products in mind. Software as a medical device was added at a later date and it is not entirely clear how the regulations relating to medical devices should be applied, either generally or in the specific case of DIY APSs.^27^ This could impact whether developers of a DIY APS could be held responsible for any harm resulting from a software defect.

Current guidance from the Medicines and Healthcare products Regulatory Agency (MHRA) states that as long as all the installation information is provided, then open-source software and uncompiled software will fall within the regulations.^28^ However, it is not clear in what circumstances a software developer that uploads and updates code for use by the DIY APS community would be classed as a ‘producer’ or ‘manufacturer’. DIY APSs development and maintenance involves numerous possible ‘producers’ or ‘manufacturers’, and the law is not clear on how these could be distilled to apportion responsibility [Correction added on 22 December 2021 after first online publication: In the preceding sentences, the words ‘and manufacturer(s)’ are added.]. For instance, in the context of 3D bioprinting, a similarly innovative technology, it has been argued that only producers of a *final finished product* can be held liable.^29^ However, identifying what counts as the final finished product (e.g. the built DIY APS or complete code plus instructions) in the DIY APS context is likely to be fraught with difficulty. Given the diffuse global and collaborative nature of the systems’ development, identifying a final producer would prove similarly problematic.

Determining whether the DIY APS software is ‘defective’ would also be a challenge. Relevant factors to consider are whether there are clear instructions for its intended use and adequate warnings to render it ‘safe’.^30^ Given that the DIY APS community has developed comprehensive development tools and instructions for use, alongside warnings that users engage at their own risk,^6^it may be argued by developers that, even if they are the ‘producer’ of a ‘product’, they have taken necessary steps to ensure their product is safe.

Whether the software developer owes a DIY APS user a duty of care has not yet been tested in the courts. *If* it was decided that there was a duty of care, the scope and standard of care would take into consideration a number of things. Significantly, these include the developer’s particular skill and whether they have acted reasonably in accordance with a body of professionals with that skill.^31^ Ordinarily, the courts could use relevant professional guidelines to shape the standard and scope of care. However, current MHRA guidance on software as a medical device does not adequately capture the DIY APS context, adding yet another layer of uncertainty here.

## 5 Conclusion

Throughout this article, we have seen that the boundaries of legal responsibility in relation to DIY APSs are unclear. In the absence of clear and settled case law or more explicit guidance from regulators and professional bodies, existing legal principles apply. In this short piece, we have indicated, in relation to DIY APSs, how the actions of clinicians, parents, manufacturers, developers, and ‘loopers’ themselves might be viewed by the law if harm were to occur from the use of one of these systems.

In summary, the (in)actions of clinicians with regard to DIY APSs fall under the usual law of clinical negligence. Applying these established principles, we indicated that clinicians are unlikely to be negligent for discussing DIY APSs with patients, providing information on DIY APSs, or even recommending DIY APS. However, discussing material risk sufficiently and representing with honesty the limits of their knowledge are central. Further, the prescribing of components (such as CGMs and insulin pumps) that may be used to loop would not be negligent if the clinician acted according to accepted standards of care. Importantly, it would be inappropriate to withhold a CGM or insulin pump from a PWD solely out of concern that they might close the loop.

With regard to children whose parents may wish to build a DIY APS, again, in the absence of more explicit guidance, existing legal principles apply. The law does not preclude DIY APS use in children, but this would need to be justified by reference to the child’s best interests and considering parental knowledge of the associated risks.

Pump manufacturers or software developers could possibly be held liable for harm caused by DIY APSs, but there are significant legal hurdles to making any such claim stick; not least the fact that users may be viewed as having assumed (at least partial) responsibility for any harm arising in virtue of having built the system themselves. The law in this area remains untested and could be clarified through future regulatory reform.

The DIY approach seems unlikely to disappear any time soon even as more commercially available models come to market. Clearer guidance from relevant bodies, particularly in relation to clinical care, should be a priority to allay concerns of clinicians and ensure appropriate care for DIY APS users.

## Footnotes

1 Currently before a medical device, including software, can be made available in the UK it must have either a UKCA (UK Conformity Assessed) mark (England, Wales and Scotland) or a CE (Conformité Européene) mark (Northern Ireland which post-Brexit is still subject to EU law in this area).

2 Within the UK there are three legal systems: England and Wales, Scotland, and Northern Ireland. Largely, the approach to the matters we reference is the same across these jurisdictions. The statutory basis and court tasked with interpretation of the law may differ but the overarching legal principles are consistent.

3 Tort law in England, Wales, and Northern Ireland, and the law of delict in Scotland.

4 Further, where a DIY user is prescribed their pump by an NHS healthcare provider there is no direct contractual relationship between the user and the manufacturer. While it is possible for a private patient who receives private healthcare to have a contractual relationship with a manufacturer from whom they purchase a pump, the re-purposing for ‘looping’ would likely fall outside the scope of any contract that is in place.

5 Inaccurate CGM data may also result in harm to the user. Here, we focus on the liability of pump manufacturers rather than CGM manufacturers. This is because pumps pose a more direct risk of harm due to their insulin delivery functionality; however, it is not clear that CGM manufacturers would not face some similar issues to pump manufacturers regarding liability.

6 E.g. ‘A Note on DIY and the ‘Open’ part of OpenAPS: This is a set of development tools to support a self-driven DIY implementation. Any person choosing to use these tools is solely responsible for testing and implementing these tools independently or together as a system…’ https://openaps.readthedocs.io/en/latest/index.html?highlight=risk#welcome-to-openaps-s-documentation.
